# Recovery from a possible cytomegalovirus meningoencephalitis-induced apparent brain stem death in an immunocompetent man: a case report

**DOI:** 10.1186/s13256-016-1034-0

**Published:** 2016-08-26

**Authors:** Theresia Monica Rahardjo, Tinni Trihartini Maskoen, Ike Sri Redjeki

**Affiliations:** 1 Anesthesiology Department, Faculty of Medicine, Maranatha Christian University, Bandung, Indonesia; 2 Anesthesiology & Intensive Care Department, Faculty of Medicine, Universitas Padjadjaran, Bandung, Indonesia

**Keywords:** Meningoencephalitis, Cytomegalovirus, Brain stem death, Immunocompetent

## Abstract

**Background:**

Recovery from cytomegalovirus meningoencephalitis with brain stem death in an immunocompetent patient is almost impossible. We present a remarkable recovery from a possible cytomegalovirus infection in an immunocompetent man who had severe neurological syndromes, suggesting brain stem death complicated by pneumonia and pleural effusion.

**Case presentation:**

A 19-year-old Asian man presented at our hospital’s emergency department with reduced consciousness and seizures following high fever, headache, confusion, and vomitus within a week before arrival. He was intubated and sent to our intensive care unit. He had nuchal rigidity and tetraparesis with accentuated tendon reflexes. Electroencephalography findings suggested an acute structural lesion at his right temporal area or an epileptic state. A cerebral spinal fluid examination suggested viral infection. A computed tomography scan was normal at the early stage of disease. Immunoglobulin M, immunoglobulin G anti-herpes simplex virus, and immunoglobulin M anti-cytomegalovirus were negative. However, immunoglobulin G anti-cytomegalovirus was positive, which supported a diagnosis of cytomegalovirus meningoencephalitis. His clinical condition deteriorated, spontaneous respiration disappeared, cranial reflexes became negative, and brain stem death was suspected. Therapy included antivirals, corticosteroids, antibiotics, anticonvulsant, antipyretics, antifungal agents, and a vasopressor to maintain hemodynamic stability. After 1 month, he showed a vague response to painful stimuli at his supraorbital nerve and respiration started to appear the following week. After pneumonia and pleural effusion were resolved, he was weaned from the ventilator and moved from the intensive care unit on day 90.

**Conclusions:**

This case highlights several important issues that should be considered. First, the diagnosis of brain stem death must be confirmed with caution even if there are negative results of brain stem death test for a long period. Second, cytomegalovirus meningoencephalitis should be considered in the differential diagnosis even for an immunocompetent adult. Third, accurate therapy and simultaneous intensive care have very important roles in the recovery process of patients with cytomegalovirus meningoencephalitis.

## Background

Cytomegalovirus (CMV) causes severe diseases in immunocompromised patients, leading to significant morbidity and mortality, either through viral reactivation from latent CMV infection or primary CMV infection. Encephalitis, pneumonitis, hepatitis, uveitis, retinitis, colitis, and graft rejection are some of the clinical syndromes that can be observed in such patients. However, little attention has been paid to CMV infection in immunocompetent patients [1, 2]. CMV infection in immunocompetent patients is usually asymptomatic because cell-mediated host immune responses prevent the development of overt CMV disease. However, some severe clinical manifestations of CMV infection in several conditions of immunocompetent patients have been reported. Critically ill patients, severity of illness markers, mechanical ventilation, bacterial pneumonia, sepsis, and transfusion may be associated with CMV infection risk [3]. Of interest, the seroprevalence of CMV is 20 to 30 % higher in non-white populations worldwide, suggesting that Asian people have a higher risk of CMV infection [4].

The most severe manifestation of CMV infection in immunocompetent patients is meningoencephalitis, which is characterized by seizures and coma as clinical manifestations. Ventriculoencephalitis is a CMV infection with brain stem involvement characterized by severe cranial nerves dysfunction and can lead to brain stem death (BSD) [5, 6]. Lung involvement can manifest as pneumonia or interstitial pneumonitis [7]. There is no existing data on the recovery process in immunocompetent patients with CMV meningoencephalitis with BSD. Some studies have only focused on CMV manifestation in immunocompromised patients. A few notable cases of CMV diseases manifested as kidney, gastrointestinal tract, and liver abnormalities, but no brain stem involvement was observed [8]. A case of CMV meningitis was previously reported, but was not accompanied by encephalitis, BSD or pneumonia with pleural effusion as complicating factors [9]. We present an exceptional case of an immunocompetent patient who recovered from BSD caused by CMV meningoencephalitis.

## Case presentation

A 19-year-old Asian man presented at our hospital’s emergency department with reduced consciousness and seizures. He had a Glasgow Coma Score of 11 to 12, and was agitated and confused during the first 2 days. He experienced two to three general tonic–clonic seizures of approximately 15 to 30 seconds’ duration each within hours of each other, and he was awake between seizures. His seizures started with stiffness in his whole body and his eyes were rolled back during seizures. His Glasgow Coma Score was reduced to 8 on the third day and he was intubated and sent to our intensive care unit (ICU). He had a continuous high fever, ranging from 39 °C to 40 °C, headache, confusion, and vomitus. His fever began to decline to 38.0 °C several hours before hospital admission. He and his family had no history of epilepsy, weakness and paralysis of limbs, drug abuse, tobacco smoking, or alcoholism.

A physical examination showed nuchal rigidity and tetraparesis with accentuated tendon reflexes. Cranial nerves and ophthalmoscopy examinations were normal. An immediate electroencephalography (EEG) showed periodic epileptogenic waves at his right temporal area and general bitemporal cortical dysfunction. These findings suggested an acute structural lesion at his right temporal area or an epileptic state, and a possible viral cause.

**Fig. 1:**
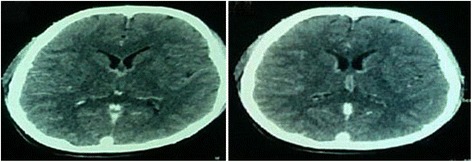
CT scan showed the first result was normal (*left*) but the second showed a smeared bright area in ependymal cells at the lower area of the third ventricle (*right*)

Evaluation of hematology showed dynamic changes of leukocytes and C-reactive protein (CRP) level during his illness. His white blood cell count and CRP reached peak level at day 70 when ventilator-associated pneumonia and pleural effusion occurred (Table 1). Coagulation parameters and a liver function test showed normal values. A cerebral spinal fluid examination showed a white blood cell count of 16/mm^3^, polymorphonucleocytes (PMN) of 13/mm^3^, mononuclear (MN) cells of 87/mm^3^, glucose level of 42/dL, and an increased protein level of 216 mg/dL, which suggested a nonspecific viral infection. Gram, India ink, and Ziehl–Neelsen stains were negative. Computed tomography (CT) scans were performed twice, on 2 September 2014 (day 6) and 22 September 2014 (day 26). The first CT scan result was normal (Fig. 1 left) but the second showed a smeared bright area in ependymal cells at the lower area of the third ventricle (Fig. 1 right). Serology tests were performed against herpes simplex virus and varicella zoster virus. These tests showed negative results for immunoglobulin (Ig) M and IgG. The possibility of human immunodeficiency virus was eliminated by a CD4 count of 750 cells/mm^3^. Different results were found in the serology test for CMV. First, a serology test showed negative results for IgM and IgG anti-CMV. A second serology test showed a borderline positive result for IgG anti-CMV with a titer of 0.9 U/mL. The last two serology tests showed positive results for IgG anti-CMV with titers of 5.0 U/mL and 3.8 U/mL. The four-fold increase in IgG anti-CMV from 0.9 U/mL to 5.0 U/mL within 8 days is an important finding (Table 2). Serial images of his thorax and clinical pulmonary infection score assessment accompanied by blood and sputum cultures were regularly performed, and confirmed a diagnosis of pneumonia in our patient. Based on his medical history, physical examinations, laboratory results, and supporting examinations, the diagnosis of CMV meningoencephalitis was made.

**Table 1:** Inflammatory markers

Markers	Day 1	Day 9	Day 39	Day 70	Day 85
White blood cells (/mm^3^)	15,800	9000	9500	18,900	7800
C-reactive protein (mg/L)	10	5	6	25	7

**Table 2:** Cytomegalovirus serology result

Cytomegalovirus	Day 9	Day 19	Day 27	Day 39
Immunoglobulin M	–	–	–	–
Immunoglobulin G	–	+ (0.9 U/mL)	+ (5.0 U/mL)	+ (3.8 U/mL)

His clinical condition deteriorated even though therapy with cefixime, acyclovir, dexamethasone, and phenytoin was administered intravenously. His cranial reflexes started to become reduced after 1 week in our ICU. On day 19, spontaneous respiration disappeared, cranial reflexes became negative, and BSD was suspected because he had no response to all brain stem tests, including an apnea test (Table 3). A vasopressor was used to maintain his hemodynamic stability. His family insisted life support should be continued indefinitely. This condition lasted for almost 2 weeks with no improvement and brain stem tests were regularly performed with negative results.

**Table 3:** Brain stem death test results

Cranial nerves	Day 21	Day 23	Day 25	Day 27	Day 30
II, III	–	–	–	–	–
V, VII	–	–	–	–	–
V, VII	–	–	–	–	–
III, VI, VIII	–	Na	Na	–	Na
IX, X	–	–	–	–	–
X	–	–	–	–	–
Apnea test	–	Na	Na	Na	Na

*Na* not applied

On day 30, he provided a vague response to painful stimuli at his supraorbital nerve. On day 35, he opened his eyes. Respiration started to appear on day 37, followed by gradual movement of his fingers. His consciousness improved from day 37, and he became fully conscious on day 50. Ganciclovir replaced acyclovir based on a four-fold increase of IgG anti-CMV serology in a test result on day 27. Other therapies on days 19 to 30 included antibiotics based on culture, corticosteroids, antibiotics, antipyretics, and antifungal agents. He also had pleural effusion and a water-sealed device was installed on day 75. A higher positive end-expiratory pressure (PEEP) on the ventilator was applied to maintain oxygenation and prevent alveoli collapse. After day 80, his respiration improved. On day 85, he was weaned from the ventilator and was able to breathe without it. He started to move his arms but his legs were still paralyzed. On day 90, he was moved from our ICU to in-patient care where he stayed for 10 days until he went home.

## Discussion

CMV is a beta herpesvirus and can cause severe neurological syndromes. This virus is found worldwide. The seroprevalence of CMV varies in different geographical areas, ranging from 30 to 100 %. It usually attacks immunosuppressed patients. CMV encephalitis in acquired immunodeficiency syndrome occurs in at least 6 % of untreated patients with advanced human immunodeficiency virus disease. CMV pneumonia occurred in 15 to 20 % of transplant recipients and patients with acquired immunodeficiency syndrome, and is associated with a mortality rate of 85 %. Most CMV infections resulted from virus reactivation rather than primary infection. CMV infection is usually asymptomatic in immunocompetent persons. Some studies have shown that CMV infection occurs in critically sick patients who are admitted to ICUs and the prevalence ranges from 0 to 35 %. It can worsen the prognosis, prolong the ICU stay, extend ventilator use, and increase nosocomial infection [1, 4, 5, 10, 11].

The diagnosis of meningoencephalitis in our patient at the beginning of his illness was based on fever, headache, a stiff neck, nausea, and vomitus as signs and symptoms of meningitis. Encephalitis was suggested by altered mental status, loss of consciousness, and cranial nerves dysfunction. The viral etiology was strengthened by supporting examinations and laboratory results [12, 13].

A tentative diagnosis of CMV meningoencephalitis was made based on serology tests and CT scan result. The first serology test in our patient on day 9 showed negative results for IgM and IgG anti-CMV. A subsequent serology test on day 19 showed a borderline positive result for IgG anti-CMV. This was followed by a positive result for IgG anti-CMV and a four-fold increase in IgG anti-CMV on day 27. The second CT scan performed on day 26 showed a smeared bright area in ependymal cells at the lower area of his third ventricle. The positive results of IgG anti-CMV and CT scan supported the diagnosis of CMV meningoencephalitis. Recovery is needed to make a definite diagnosis.

CMV reactivation, but not primary infection, was suggested in our patient based on serology method with a positive result of IgG anti-CMV, a four-fold increase in IgG anti-CMV, and a negative result for IgM anti-CMV. The negative result for IgM anti-CMV may have been due to unformed IgM or IgM that was produced but did not reach a detectable level. The immunocompetent status of our patient could also have affected the level of IgM anti-CMV formed. The negative result for IgG anti-CMV until day 19 is related to the time required for IgG production, known as the infection window period. This condition can occur in some viruses and is affected by the immunological status of the patient and immunosuppressive drugs.

The subacute type of CMV infection in our patient, called ventriculoencephalitis, was supported by severe cranial nerve dysfunction, leading to BSD. All his cranial reflexes disappeared on day 19. All brain stem tests, including an apnea test that was performed on day 21, showed no responses. These findings supported the suspected condition of BSD. This condition was consistent with the results of a CT scan and serology results on day 26 and day 27, respectively. A second complete BSD examination could not be performed because the patient’s family refused a second apnea test. Therefore, the diagnosis of BSD could not be made. Our patient was on a ventilator and used a vasopressor with no improvement for almost 2 weeks. However, his family refused withdrawal of life support. During his illness, he was administered antivirals, corticosteroids, antibiotics, antipyretics, and antifungal medication [14].

There are no uniform guidelines available for diagnosis of CMV infection in such critically ill, but immunocompetent, patients. An ideal diagnostic test should be able to detect active CMV infection and differentiate it from CMV disease. CMV infection is defined as isolation of virus (viral culture), or detection of CMV proteins (pp65) or nucleic acid by a polymerase chain reaction from blood or other clinical samples. CMV disease is confirmed by clinical findings suggestive of organ involvement, as well as a demonstration of the virus by viral isolation, histopathological testing, immunohistochemical analysis, or *in situ* hybridization from the relevant clinical sample obtained from the site of involvement. Detection of CMV by polymerase chain reaction alone is insufficient for confirming CMV disease. Conventional methods for the diagnosis of CMV infection or disease are serology, including CMV-specific antigen and antibody detection, which were used in our patient, viral isolation by viral culture, and the molecular method for detection of viral DNA from blood and other clinical specimens [15–17].

Antiviral medication was changed from acyclovir to ganciclovir on day 27 after the diagnosis of CMV meningoencephalitis was supported by a serologic result. All brain tests, except for the apnea test, were performed on the same day and showed negative results. An extraordinary event occurred in our patient on day 30, after 4 days of ganciclovir treatment. We observed a vague response to painful stimuli at his supraorbital nerve when routine brain stem tests were performed. The response became apparent and he opened his eyes on day 35. However, respiration was still absent at this time and started to appear as small respiratory efforts on day 37, followed by motor activity at his fingers. He became fully conscious on day 50. He started to breathe without the ventilator on day 85 and moved out of our ICU on day 90.

Ventilator-associated pneumonia and pleural effusion act as complicating factors that worsen and prolong the recovery process. Our patient was able to breathe on his own with continuous positive airway pressure of 5 mmHg on day 66, but his respiration started to decrease within the next week, accompanied by fever. His clinical pulmonary infection score increased by more than 6. Major pleural effusion was detected on day 75 and a water-sealed device was applied on the same day. His fever began to resolve after replacement of antibiotics on day 69 and his respiration started to improve. Cultures were regularly performed every 5 days and antibiotics were used according to the result. Antifungal medication was provided because our patient had a Candida score of greater than 3 [18].

### Study limitation

This case report has a few limitations. First, the diagnosis of BSD could not be made because our patient’s family did not give permission for a second apnea test. However, changes in result from a first to a second BSD test are small. Therefore, even though a second BSD test could not be performed, it was almost impossible that our patient would regain brain stem function by the time of a second test after the negative results of the first BSD test [19]. Second, there is the limitation that supportive and advance examinations were not performed because of financial reasons. Serology tests for IgM and IgG anti-CMV could only be performed four times, a CT scan two times, and EEG once. Magnetic resonance imaging was not performed. Magnetic resonance imaging is superior to CT scans in evaluating central neural system infections, including meningoencephalitis, but this limitation can be overcome by a positive result in a CT scan and serology test.

## Conclusions

In conclusion, this case report shows an unusual recovery process from CMV meningoencephalitis in an immunocompetent patient with BSD. Even though there has been advancement in our understanding of the relationship between CMV infection with brain stem involvement and immunocompetence in a patient, our case is unusual. A diagnosis of BSD must be confirmed with extreme caution, even if all BSD tests show negative results for a long time. CMV meningoencephalitis should also be considered in the differential diagnosis in immunocompetent patients whose cerebral spinal fluid results show a suspected viral infection. Furthermore, accurate therapy and simultaneous intensive care are important in a patient’s recovery process. The availability of diagnostic testing also accelerates determination of diagnosis, and this will affect treatment and prognosis.

## Abbreviations

BSD: Brain stem death
CMV: Cytomegalovirus
CRP: C-reactive protein
CT: Computed tomography
EEG: Electroencephalography
ICU: Intensive care unit
Ig: Immunoglobulin
